# Polymicrobial Pituitary Abscess Predominately Involving* Escherichia coli* in the Setting of an Apoplectic Pituitary Prolactinoma

**DOI:** 10.1155/2016/4743212

**Published:** 2016-02-23

**Authors:** Norman Beatty, Luis Medina-Garcia, Mayar Al Mohajer, Tirdad T. Zangeneh

**Affiliations:** Division of Infectious Diseases, Department of Medicine, University of Arizona College of Medicine, Banner-University Medical Center, 1501 N. Campbell Avenue, Tucson, AZ 85724, USA

## Abstract

Pituitary abscess is a rare intracranial infection that can be life-threatening if not appropriately diagnosed and treated upon presentation. The most common presenting symptoms include headache, anterior pituitary hypofunction, and visual field disturbances. Brain imaging with either computed tomography or magnetic resonance imaging usually reveals intra- or suprasellar lesion(s). Diagnosis is typically confirmed intra- or postoperatively when pathological analysis is done. Clinicians should immediately start empiric antibiotics and request a neurosurgical consult when pituitary abscess is suspected.* Escherichia coli* (*E. coli*) causing intracranial infections are not well understood and are uncommon in adults. We present an interesting case of an immunocompetent male with a history of hypogonadism presenting with worsening headache and acute right eye vision loss. He was found to have a polymicrobial pituitary abscess predominantly involving* E.   coli* in addition to* Actinomyces odontolyticus* and* Prevotella melaninogenica* in the setting of an apoplectic pituitary prolactinoma. The definitive etiology of this infection was not determined but an odontogenic process was suspected. A chronic third molar eruption and impaction in close proximity to the pituitary gland likely led to contiguous spread of opportunistic oral microorganisms allowing for a polymicrobial pituitary abscess formation.

## 1. Introduction 

Pituitary abscess is a rare intracranial infection that accounts for 0.24–0.6% of all pituitary lesions [1]. It can be life-threatening if not appropriately diagnosed and treated upon presentation. Immediate administration of empiric antibiotics and request for a neurosurgical consultation are recommended when pituitary abscess is suspected. Common presenting symptoms include headache, anterior pituitary hypofunction, and visual field disturbances [1, 2]. Brain imaging with either computed tomography or magnetic resonance imaging typically displays intra- or suprasellar lesion(s). Diagnosis is typically confirmed intra- or postoperatively when pathological and microbiologic analysis is done. Pituitary abscess can be either primary (occurs in normal gland) or secondary (occurs in preexisting pathology) [2]. The exact etiology of secondary pituitary abscess is not well understood. It has been postulated that pituitary abscess formation occurs through either hematogenous and/or direct contiguous spread by an adjacent infectious process [2]. Intracranial infections caused by* E. coli* are extremely rare among the adults [3]. It still remains unclear why only a limited amount of intracranial* E. coli* infections have been described in adults when bacteremia is so common. We present a unique case of an immunocompetent male with a history of hypogonadism presenting with worsening headache and acute right eye vision loss. He was found to have a polymicrobial pituitary abscess predominantly involving* E. coli* in addition to* Actinomyces odontolyticus* and* Prevotella melaninogenica* in the setting of an apoplectic pituitary prolactinoma.

## 2. Case Presentation 

A 24-year-old male presented to the emergency department with acute right eye blindness, agitation, and altered mental status of one-day duration. His wife who provided the initial history accompanied the patient. He had been initially evaluated by a neurologist for new-onset headaches in an outside facility. He was referred to our institution of further evaluation. During their trip, the patient had sudden loss of vision in his right eye and en-route to our facility he became altered and lost consciousness. Intravenous fluid boluses were initiated and the patient was evaluated in the emergency department.

According to the wife, the patient was otherwise healthy with no recent illnesses. In the previous month, he was found to have a low testosterone level along with bilateral microorchidism. His medications consisted of oral testosterone prescribed to him in Mexico. He had a two-year history of intermittent odontogenic pain mildly relieved with over-the-counter analgesics. The patient was allergic to amoxicillin. He consumed alcohol occasionally and later denied use of tobacco or illicit drugs. Family history was positive for hypertension but no known familial conditions. He was employed at a local pizza restaurant and lived in Arizona. He frequently traveled to Mexico. He was sexually active with his wife and they did not use barrier protection. There were no reported known sick contacts.

**Figure 1:**
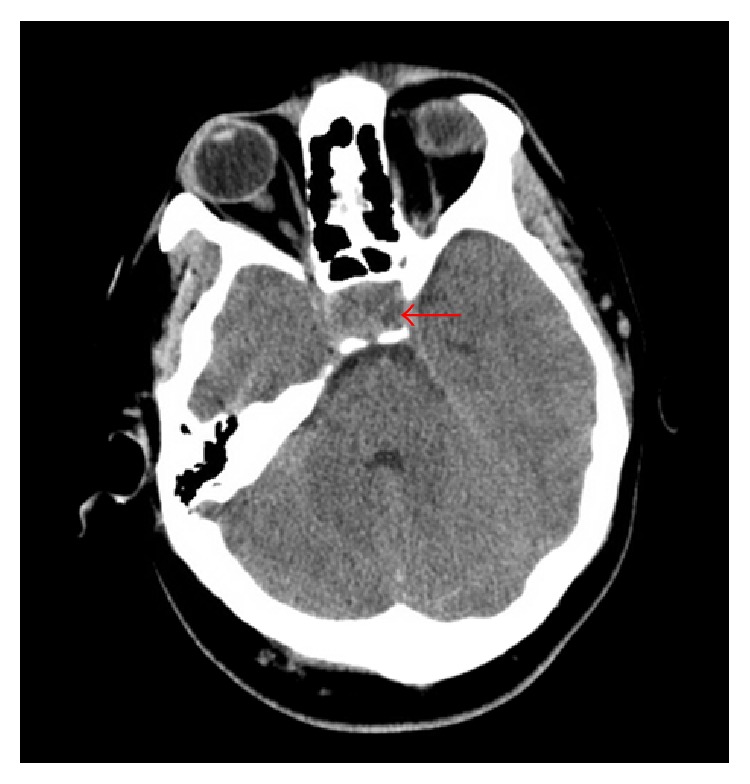
Noncontrast head CT. Red arrow indicating intra-/parasellar mass.

**Figure 2:**
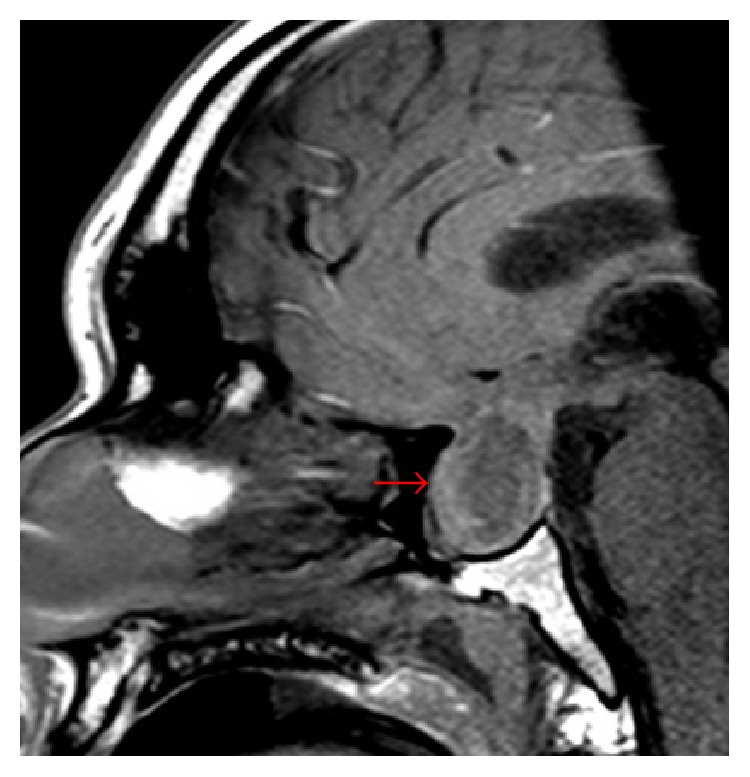
T1 SAG SELLA (MRI brain with and without contrast). Red arrow indicating large pituitary mass.

**Figure 3:**
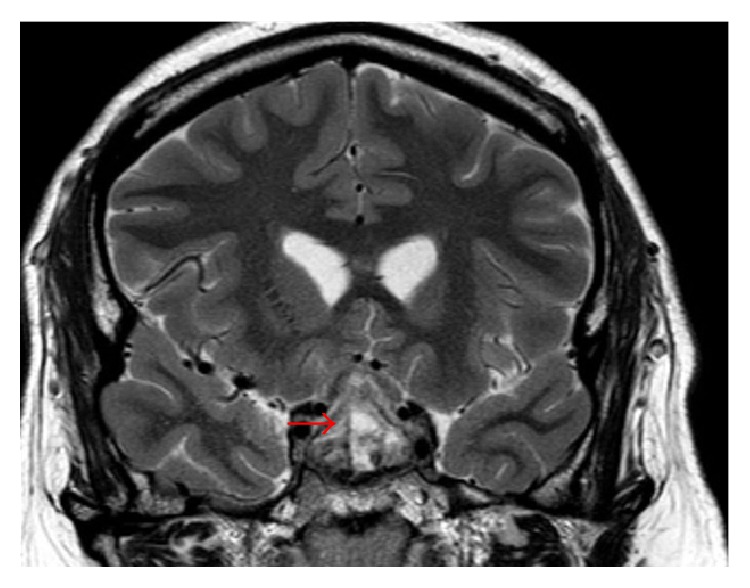
T2 COR SELLA (brain MRI with and without contrast). Red arrow indicating pituitary mass with evidence of internal hemorrhage.

**Figure 4:**
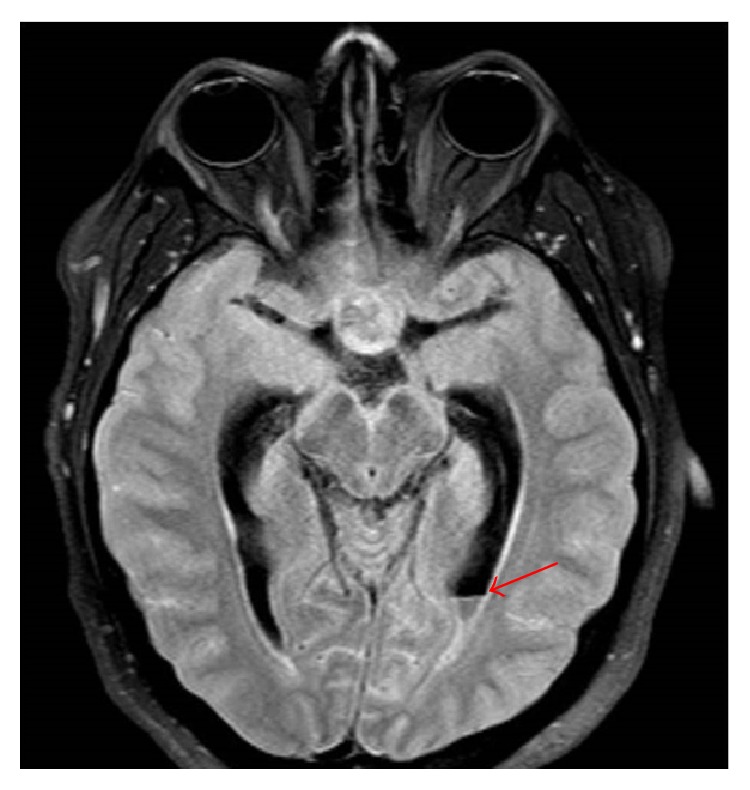
T1 AX FLAIR (brain MRI with and without contrast). Red arrow indicating debris layering in the left ventricle.

Initial vital signs in the emergency department revealed a heart rate of 110 beats/min, temperature of 36.8°C, respiratory rate of 21 breaths/min, and blood pressure of 152/64 mmHg. On physical exam, the patient was intermittently conscious, agitated at times, and arousable to sternal rub when somnolent. His right pupil was not reactive to light and was fixed at 8 mm. Lungs were clear and abdomen examination was benign. Gynecomastia was absent. Pertinent genitourinary examination findings revealed testicular size of 3 × 2 × 1 cm, which was grossly smaller than the average postpubescent testicular size. Neurological exam was limited, as he would not follow commands. All four extremities had unpurposeful movements with bilateral flexor plantar reflexes. The score of the Glasgow coma scale (GCS) was 8 (E2V2 M4) on admission prompting intubation of his trachea for airway protection. The initial impression was that he had sepsis secondary to a central nervous infection such as bacterial meningitis. Blood cultures were drawn prior to the administration of intravenous (IV) ceftriaxone and vancomycin. Noncontrast head computed tomography (CT) revealed a suspicious sellar/parasellar mass with evidence of abnormal material in the subarachnoid space without intracranial hemorrhage (Figure 1). Brain magnetic resonance imaging (MRI) with and without contrast displayed a pituitary mass (Figure 2) with internal hemorrhage (Figure 3). Diffuse leptomeningeal and subependymal enhancement with debris layering in both lateral ventricles (Figure 4) was also noted. These findings were consistent with pituitary macroadenoma and apoplexy. Potential superimposed meningitis and ventriculitis could not be ruled out. Initial laboratory work (Tables 1 and 2) revealed pertinent findings of a total white blood cell count (WBC) 16.1 × 1000/*μ*L with neutrophilic-predominance and a serum lactic acid of 6.0 mMol/L.

**Figure 5:**
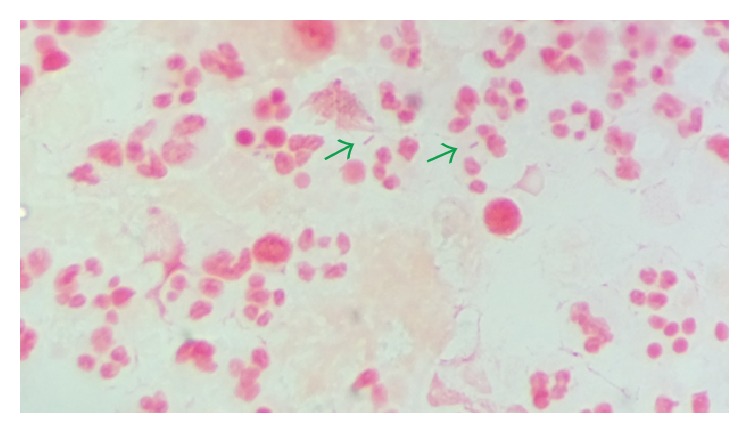
Pituitary aspirate Gram stain. Green arrows indicating Gram-negative bacilli.

Emergent neurosurgical, otolaryngology, and infectious diseases consultations were requested. He was administered a single dose of IV hydrocortisone and was immediately taken to the operating room. Endoscopic endonasal pituitary decompression and resection of a pituitary mass were successful. Tissue samples were sent for culture and pathology for review. Intraoperative reports described necrotic tissue with purulent fluid noted grossly once the dural space was entered. An external ventricular drain (EVD) was placed during surgery for monitoring of cerebrospinal fluid (CSF) pressures due to the possibility of hydrocephalus. The patient was noted to have fever postoperatively at 38.2°C. His antibiotic regimen was changed to IV meropenem once admission blood culture revealed growth of* E. coli.* Gram stain analysis from the pituitary aspirates revealed the presence of Gram-negative bacilli (Figure 5) with early growth of* E. coli* on culture. Additional colonies of* Actinomyces odontolyticus* and* Prevotella melaninogenica* were subsequently isolated from the pituitary aspirate. Over a seven-day period, our patient continued to experience undulating low-grade fevers despite adequate intravenous antibiotic administration. Urine and blood cultures were repeatedly negative. However, CSF cultures were persistent for* E. coli* with similar antibiotic susceptibility patterns to the pituitary and original blood isolates. The presence of multiple CSF cultures growing* E. coli* prompted removal of the EVD out of concern for contamination. A follow-up lumbar puncture confirmed eradication of* E. coli* from the CSF (Table 3). The patient remained afebrile after EVD removal and leukocytosis resolved. Due to his reported amoxicillin allergy, a graded-dose challenge of IV ampicillin/sulbactam was attempted and initially tolerated. Unfortunately, the patient later developed a diffuse morbilliform drug eruption; thus, eight weeks of IV meropenem was recommended.

**Figure 6:**
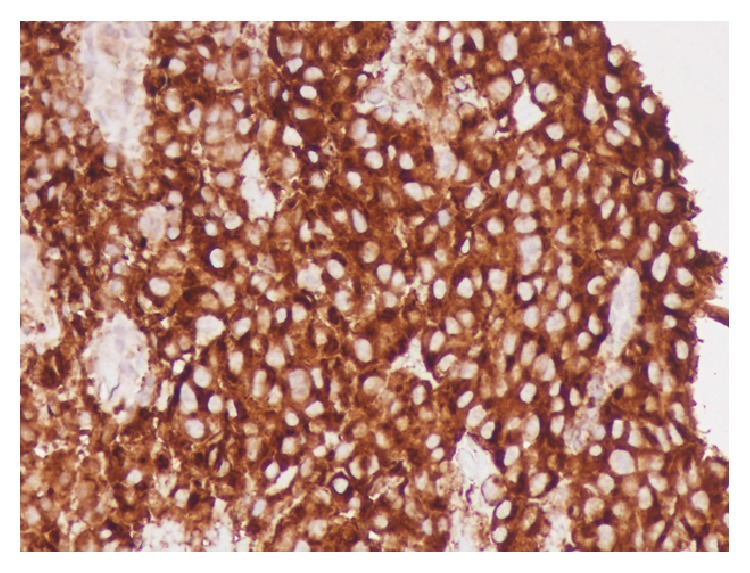
Prolactin stain of pituitary mass confirms prolactinoma.

The pituitary adenoma was positive for a prolactinoma after staining (Figure 6) which correlated with the serum hormone levels found on admission (Table 4). Our patient was initially managed with stress doses of hydrocortisone that were eventually tapered off after morning cortisol levels were found to be normal. Cabergoline and levothyroxine were started for the management of continued hyperprolactinemia and subclinical hypothyroidism. On the day of discharge, he reported improved vision in his right eye with an occasional “floater.” Four weeks after discharge, he was doing well when seen in clinic. In total, he received eight weeks of IV meropenem and one additional month of oral doxycycline for extended treatment of intracranial* Actinomyces odontolyticus* infection. At a six-month follow-up, he did not exhibit any clinical evidence of reemerging infection but did endorse some lateral field visual deficits. Repeat MRI pituitary imaging yielded no new changes and some lateral field visual deficits. as an outpatient with surveillance imaging.

## 3. Discussion

*E. coli* intracranial infections in adults are rare and poorly understood [3]. Prior cases involving intracranial infections due to* E. coli* were usually associated with a poor prognosis and minimal response to antibiotics [3]. A recent literature search described only one other case involving an* E. coli* pituitary abscess in the setting of a pituitary prolactinoma [4]. Our case is unique because our patient had concurrent* E. coli* bacteremia. It still remains unclear why the hematogenous spread of* E. coli* to the central nervous system is so rare among adults [3]. The etiology of pituitary abscess formation is poorly understood and the majority of patients have no predisposing factors [5]. Two major factors associated with pituitary abscess formation are prior surgery in this region and an existing pituitary lesion [5]. Our patient did not have a history of prior surgery but an active prolactinoma. In most cases, purulent fluid is almost always present during surgical intervention but only 10% will yield growth of an organism [5]. The most common bacterial causes of pituitary abscess are streptococci and staphylococci species [6]. As in our case, it is important to keep in mind that pituitary apoplexy may be a viable source of microbial seeding from hematogenous or contiguous spread leading to pituitary abscess formation [5, 6]. The most common presenting symptoms of pituitary abscess are headache and visual abnormalities [6]. When pituitary abscess is suspected, prompt neurosurgical intervention and broad spectrum antibiotic administration are important key elements to initial treatment [5, 6]. The mortality rate of a pituitary abscess was once reported within 30–50% but outcomes have since improved significantly [5, 6]. A recent review of the literature has indicated that the overall mortality was estimated to be 3% [5]. On the other hand, Gram-negative bacillary meningitis and/or concomitant ventriculitis have been shown to carry unfavorable outcomes. In these cases, intraventricular antibiotic administration has been shown to decrease mortality and morbidity rates, especially in those who are refractory to intravenous antibiotic treatment [7]. Our patient responded well to conventional intravenous and surgical therapies and did not require intraventricular antibiotics.

**Figure 7:**
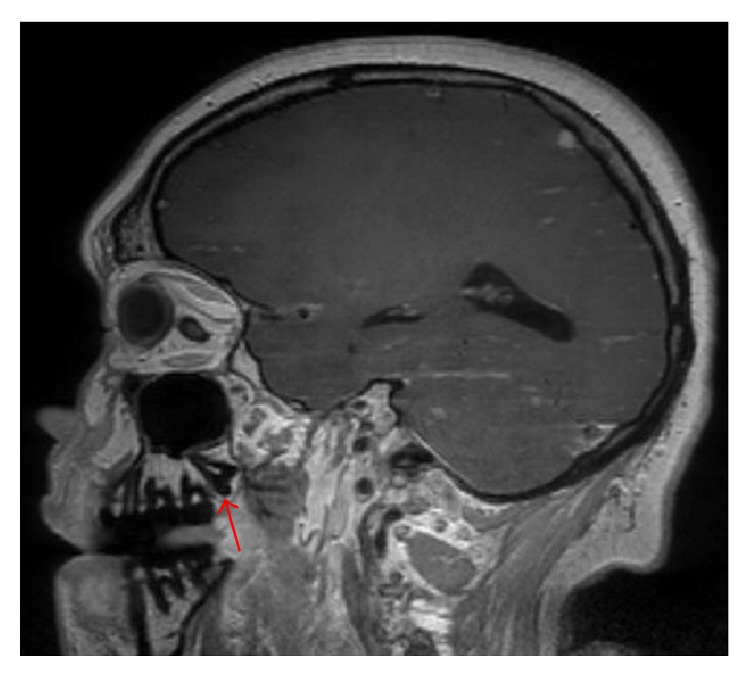
Impacted upper right third molar seen on MRI in close proximity to diseased pituitary gland.

A definitive etiology of our patient's source of infection is yet to be determined. Thorough investigations failed to provide a conclusive explanation for his polymicrobial pituitary abscess. It is likely that this pituitary abscess was the source of his* E. coli* bacteremia since the blood isolates had similar antibiotic susceptibility patterns to those grown on the pituitary and CSF cultures. However, genetic analysis of the isolated* E. coli* strains was not done; thus, we can only infer that they were similar based on their antibiotic susceptibilities and the overall clinical picture.* E. coli* is considered normal flora of the gastrointestinal tract and CT imaging of the abdomen and pelvis did not reveal an obvious intra-abdominal source. Urinalysis was also negative which ruled out a urinary source.* E. coli* odontogenic infections are also documented and can often lead to head and neck abscess formation [8]. Further history revealed that the patient had experienced intermittent pain involving both upper third molars for several years. Further physical examination of his oral cavity showed eruption and impaction of both upper third molars with surrounding mucosal erythema. This impaction could be seen on brain MRI (Figure 7) which was in close proximity to the diseased pituitary gland. However, no definitive fluid collection or abscess was seen in the region of these third molars. Entertaining the fact that* E. coli* was found in the gastrointestinal tract and subsequent perianal region, we asked the patient whether he participated in oral sex. The patient did endorse this exposure risk. We believe that oral sex practices in this patient has introduced* E. coli* to his oral flora allowing this microorganism to opportunistically invade a nearby diseased pituitary gland leading to abscess formation. This is further supported by the fact that* Actinomyces odontolyticus* and* Prevotella melaninogenica* both grew from the pituitary aspirate. These two microorganisms are normal flora of the oral cavity and are known to cause periodontal disease and dental caries [9–11]. In conclusion, this is a unique case, which highlights a rare intracranial infection with an unlikely host of microorganisms. Odontogenic conditions, such as impaction, may lead to more serious head and neck infections with opportunistic microorganisms found in the oral flora of susceptible patients. In the right clinical setting, molar impaction or periodontal disease may allow for mucosal barrier breakdown and the possible microinvasion of the pituitary gland allowing for abscess formation. Based on this information, it was recommended that our patient visit an oral surgeon upon discharge to have these impacted third molars surgically removed.

## Figures and Tables

**Table 1:** Initial laboratory findings.

Complete blood count	Results	Reference ranges
Hemoglobin	14.8	13.5–17.5 g/dL
Hematocrit	44.3	40.0–51.0%
White blood cell count	**16.1**	3.4–10.4 × 1000/*μ*L
Platelet count	210	150–425 × 1000/*μ*L
Neutrophils (absolute)	**14.10**	1.80–7.00 × 1000/*μ*L
Lymphocyte (absolute)	**0.70**	1.00–4.80 × 1000/*μ*L
Monocyte (absolute)	**1.30**	0.30–1.00 × 1000/*μ*L
Eosinophil (absolute)	0.00	0.00–0.50 × 1000/*μ*L
Basophil (absolute)	0.00	0.00–0.10 × 1000/*μ*L

**Table 2:** 

Complete metabolic panel	Results	Reference ranges
Sodium	138	136–145 mMol/L
Potassium	3.2	3.5–5.1 mMol/L
Chloride	102	101–111 mMol/L
Carbon dioxide, total blood	**18**	20–29 mMol/L
BUN	9	9–26 mg/dL
Creatinine	0.8	0.7–1.3 mg/dL
Glucose	**264**	70–105 mg/dL
Albumin	4.0	3.5–5.0 g/dL
Total bilirubin	1.3	0.2–1.2 mg/dL
Alkaline phosphatase	75	40–115 IU/L
ALT	17	0–55 IU/L
AST	12	5–34 IU/L
C-reactive protein	**13.15**	<0.6 mg/dL
Lactate, venous	**6.0**	0.5–2.2 mMol/L

**Table 3:** 

Culture source	Results
Hospital day 1	
Blood (admission)	*E. coli*
Pituitary aspirate	*E. coli, A. odontolyticus, P. melaninogenica*
Hospital day 2	
Blood	No growth
Urine	No growth
CSF (EVD)	*E. coli*
Hospital day 5	
Blood	No growth
CSF (EVD)	*E. coli*
Hospital day 9	
Blood	No growth
CSF (EVD)	*E. coli*
Hospital day 10	
CSF (lumbar tap)	No growth

CSF: cerebrospinal fluid; EVD: extraventricular drain.

**Table 4:** 

Hormone serum levels	Results	Reference ranges
Prolactin	**301.0**	3.5–19.4 ng/mL
Thyroid stimulating hormone	**0.07**	0.35–4.00 *μ*IU/mL
Free T4	0.7	0.7–1.5 ng/dL
Cortisol (morning)	13.4	3.2–38.4 mcg/dL
